# Impact on bias mitigation algorithms to variations in inferred sensitive attribute uncertainty

**DOI:** 10.3389/frai.2025.1520330

**Published:** 2025-03-06

**Authors:** Yanchen Wang, Lisa Singh

**Affiliations:** ^1^Department of Computer Science, Georgetown University, Washington, DC, United States; ^2^Department of Computer Science and School of Public Policy, Georgetown University, Washington, DC, United States

**Keywords:** inferred sensitive attribute, machine learning fairness, bias mitigation, demographic inference, social media

## Abstract

Concerns about the trustworthiness, fairness, and privacy of AI systems are growing, and strategies for mitigating these concerns are still in their infancy. One approach to improve trustworthiness and fairness in AI systems is to use bias mitigation algorithms. However, most bias mitigation algorithms require data sets that contain sensitive attribute values to assess the fairness of the algorithm. A growing number of real world data sets do not make sensitive attribute information readily available to researchers. One solution is to infer the missing sensitive attribute information and apply an existing bias mitigation algorithm using this inferred knowledge. While researchers are beginning to explore this question, it is still unclear how robust existing bias mitigation algorithms are to different levels of inference accuracy. This paper explores this question by investigating the impact of different levels of accuracy of the inferred sensitive attribute on the performance of different bias mitigation strategies. We generate variation in sensitive attribute accuracy using both simulation and construction of neural models for the inference task. We then assess the quality of six bias mitigation algorithms that are deployed across different parts of our learning life cycle: pre-processing, in-processing, and post-processing. We find that the disparate impact remover is the least sensitive bias mitigation strategy and that if we apply the bias mitigation algorithms using an inferred sensitive attribute with reasonable accuracy, the fairness scores are higher than the best standard model and the balanced accuracy is similar to that of the standard model. These findings open the door for improving fairness of black box AI systems using some bias mitigation strategies.

## 1 Introduction

In recent years, we have seen the proliferation of powerful state-of-the-art AI systems - from targeted recommendation systems to health assistants to more general tools like ChatGPT. While these systems benefit us in many ways, researchers (and the general public) are becoming more concerned about the trustworthiness and fairness of these black-box AI systems (Li and Zhang, 2023; Zhang et al., 2023; Wang and Singh, 2024). To measure and improve the trustworthiness and fairness of machine learning tools, different fairness metrics and bias mitigation strategies have been developed (Mehrabi et al., 2021; Pessach and Shmueli, 2022). A number of open access fairness toolkits have also emerged to help researchers measure different types of fairness and test different bias mitigation strategies, e.g. AI Fairness 360 by IBM (Bellamy et al., 2019), Fairlearn by Microsoft (Bird et al., 2020), and the What-if tool by Google (Wexler et al., 2019). Not surprisingly, the bias mitigation strategies in these toolkits require the data to have values for any sensitive attribute(s). In general, most of the metrics and bias mitigation algorithms require sensitive (or protected) attribute information to measure and fix existing bias (Hu et al., 2021).

Unfortunately, in some real world data sets, sensitive attributes are not available to researchers, or they may be difficult or costly to obtain (Veale and Binns, 2017; Holstein et al., 2019). For example, researchers may use posts from social media platforms like Reddit or Twitter/X to try to understand polarization, misinformation, public opinion, etc. Sensitive demographic features about these users are not only difficult and costly to collect, but also inconsistent across platforms. Singh et al. (2020) analyzed required demographic information across some popular social media platforms, including Twitter/X, Facebook, TikTok, LinkedIn, and Snapchat, and found large variability in the type and amount of demographic information required to create an account on these platforms. For example, gender and birthdate were required to create an account on Facebook, but neither were required to have an account on LinkedIn. This inconsistency in the requirements of sensitive demographic features means that even if sensitive demographic data are made available to researchers (by user consent or by platform agreement), much of it will still contain missing values. In this paper, we refer to the lack of a ground truth sensitive attribute as the *missing sensitive attribute problem*.

One approach to for dealing with a missing sensitive attribute is to infer it While different methods for inferring the sensitive attribute have been proposed, limited knowledge exists about how the effectiveness of different bias mitigation strategies changes as the uncertainty of the inferred sensitive attribute varies, i.e., when the accuracy is higher or lower. Our goal is to fill this gap for binary sensitive attributes, specifically binary versions of gender and race.^1^

To accomplish this, we use both a combination of simulation analysis and different neural models on different data sets to investigate how the accuracy of an inferred sensitive attribute impacts the performance of different bias mitigation algorithms on three data sets, two traditional fairness data sets and one social media data set. We use a simulation study to analyze the traditional fairness data sets and we construct multiple neural models with varying accuracies to analyze the social media data set. By using both simulation and neural model construction on different types of data, we hope to better understand the sensitivity of bias mitigation algorithms to errors in the inferred sensitive attribute, and how much variation exists across different data sets and different mitigation methods. Ultimately, our goal is assess the quality of different bias mitigation strategies with respect to different levels of accuracy in the inferred sensitive attribute for general classification tasks and compare classification accuracy and fairness by assessing their sensitivity to different levels of error in the inferred sensitive attribute.

While we are not aware of a study designed like ours, there are some similar studies. Ghosh et al. (2021a) investigate how uncertainty in demographic inference impacts fairness guarantees in ranking algorithms. Awasthi et al. (2020) study how imperfect group information affects performance of post-processing bias mitigation methods. In addition to our research question being different, we focus on a broader set of bias mitigation algorithms, allowing this study's results to be applicable to a broader set of machine learning tasks. We also conduct a more detailed case study using social media data that incorporates demographic inference algorithms designed for social media within our analysis.

Our main contributions can be summarized as follows. (1) We formally define the missing sensitive attribute problem and present a methodology for understanding the impact of an inferred sensitive attribute on different bias mitigation strategies. (2) Using a simulation study, we explore how the accuracy of the inferred sensitive attribute impacts the performance (in terms of balanced accuracy and fairness) of various bias mitigation algorithms applied at different points in the machine learning life cycle (pre-processing, in-processing and post-processing), enabling us to better understand the overall impact of an inferred sensitive attribute with different levels of accuracy on bias mitigation strategies. We find that different bias mitigation algorithms have varying levels of sensitivity, and across all bias mitigation strategies, using an inferred sensitive attribute with reasonable accuracy has less bias than using a classifier that does not employ bias mitigation at all. (3) To compliment the simulation study, we also conduct a case study with social media data where we use multiple existing demographic inference models to infer the sensitive attribute, giving us a range of different sensitive values. We then apply different bias mitigation algorithms using the inferred knowledge. Similar to the simulation results, we find that using an inferred sensitive attribute results in more fair models than using the original biased model. (4) We release our code so that other researchers can advance research in this area.^2^

The remainder of this paper is organized as follows. We discuss related literature in Section 2. In Section 3, we formulate the problem. Section 4 describes our methodology. Our experimental design is discussed in Section 5, followed by the empirical evaluation in Section 6. Conclusions and future directions are presented in Section 7.

## 2 Related literature

We begin this section by discussing bias mitigation algorithms that require a sensitive attribute (Section 2.1). We then discuss the missing sensitive attribute literature (Section 2.2).

### 2.1 Bias mitigation algorithms

To correct bias, a number of bias mitigation algorithms have been proposed. For ease of exposition, we categorize the mitigation algorithms based on when they are applied in the machine learning life cycle: pre-processing, in-processing, and post-processing.

Pre-processing mechanisms attempt to fix the input data in order to improve fairness and train machine learning models using input data that are more fairly distributed with respect to sensitive attribute information (Feldman et al., 2015; Wang and Singh, 2021; Kamiran and Calders, 2012; Mehrabi et al., 2019; Brunet et al., 2019; Calmon et al., 2017; Romano et al., 2020; Li and Vasconcelos, 2019; Krasanakis et al., 2018). Approaches include: resampling, reweighting, and updating feature values. Resampling is used to remove selection bias and representation bias in the training data by using uniform resampling in under-represented groups and randomly dropping samples in over-represented groups (Wang and Singh, 2021; Romano et al., 2020; Li and Vasconcelos, 2019). Reweighting changes the sample weight during classifier training. It assigns higher weights for under-represented groups and lower weights for over-represented groups (Kamiran and Calders, 2012; Krasanakis et al., 2018). Disparate impact remover uses an approach that updates feature values to improve group fairness. It decreases the earth mover's distance between feature distributions of different sensitive groups so that they are the same across sensitive groups (Feldman et al., 2015).

In-processing mechanisms change the machine learning model during the training process, e.g., adding fairness constraints or fairness regularization components into the optimization problem (Kamishima et al., 2012; Zafar et al., 2017; Berk et al., 2017; Vapnik and Izmailov, 2015; Zhang et al., 2018; Padh et al., 2021; Wadsworth et al., 2018; Beutel et al., 2017; Xu et al., 2019; Manisha and Gujar, 2018; Wu et al., 2018). Researchers have proposed variants of adversarial debiasing. In this adversarial learning paradigm, an adversary tries to predict the sensitive attribute from the model. To prevent the adversary from predicting the sensitive attribute correctly, the algorithm changes the inference model to maximize the predictor's ability to infer the outcome, while minimizing the adversary's ability to infer the sensitive attribute by changing the weights of classifier's parameters that contain information about the sensitive attribute (Zhang et al., 2018; Wadsworth et al., 2018; Beutel et al., 2017; Edwards and Storkey, 2015; Xu et al., 2019). Another in-process approach adds regularization and changes constraints during model optimization (Manisha and Gujar, 2018; Agarwal et al., 2018; Wu et al., 2018). For example, Agarwal et al. (2018) present exponentiated gradient reduction. Their approach breaks down fair classification into a series of cost-sensitive classification problems and returns a classifier with the lowest empirical error predicting the outcome subject to the specific fairness constraints. Wu et al. (2018) propose a convex optimization with fairness constraints that can be directly incorporated into loss function optimization and they prove that the fairness constraints are upper-bounded by convex surrogate functions. This approach differs from previous work because it does not make use of surrogate constraints that may not be a reasonable estimate of the original fairness constraint. Manisha and Gujar (2018) propose FNNC, a new method to convert existing fairness metrics including disparate impact, demographic parity, and equalized odds into differentiable loss functions that can be easily adapted into any loss function so that the loss function can optimize for both accuracy and fairness.

Post-processing mechanisms change predicted labels after the model is trained. These approaches modify the results of a trained classifier to ensure fair prediction results based on the sensitive attributes (Pleiss et al., 2017; Noriega-Campero et al., 2019; Hardt et al., 2016; Kamiran et al., 2012). For example, thresholding is a popular post-processing mechanism where changes are made to the probability threshold for decision making based on the sensitive attribute. Hardt et al. (2016) introduce threshold optimizer, a strategy that utilizes the decision probability from the classifier. For example, in binary classification, a classifier returns the probability of the sample belonging to the positive and negative classes. In conventional prediction tasks, the probability threshold is set to be 0.5 for determining the final class label, e.g., if the prediction probability of an example is less than 0.5, the approach labels it as being the negative class. However, instead of 50/50, this method determines different probability thresholds for each subgroup. Similarly, Kamiran et al. (2012) modify the prediction labels for individuals that are close to the decision boundary. If we use 50/50 as the decision boundary, the prediction labels on individuals with prediction probability near the boundary (for example within 5%) will be flipped. Individuals in the privileged group will receive a negative outcome and individuals in the unprivileged group will receive a positive outcome. Another post-processing mechanism is calibration. It adjusts the probability outputs of a model so that the proportion of predicted positive outcomes is the same as the actual outcomes across all sensitive attribute groups. To maintain the calibration, Pleiss et al. (2017) propose an approach to randomly select individuals from the unprivileged group who receive negative outcomes and change their prediction labels from negative to positive. This approach can maintain the calibration and satisfy the equal opportunity requirement on fairness. Noriega-Campero et al. (2019) further improve this algorithm. Instead of the randomization approach, they propose using an information cost based as the difficulty associated with classifying an individual. If an individual is easier to classify, i.e., further away from the decision boundary, the information cost is smaller and if an individual is closer to the decision boundary, the information cost is higher since more information is needed from the individual to make the decision. To improve fairness, the algorithm changes prediction labels only on individuals with a high information cost.

### 2.2 The missing sensitive attribute problem

All bias mitigation algorithms in the popular toolkits such as AI Fairness 360 (Bellamy et al., 2019), Fairlearn (Bird et al., 2020), and the What-if tool (Wexler et al., 2019) require ground truth information about the sensitive attribute. Weerts et al. (2024) introduce guidelines and opportunities for fairness aware automated machine learning, highlighting the importance of having a sensitive attribute to measure fairness and improve fairness using existing bias mitigation algorithms.

However, the ground truth sensitive attribute is not always available. There are three approaches to tackling this missing sensitive attribute problem: approximating the sensitive attribute, privately sharing sensitive attribute information, and changing the bias mitigation algorithm to not require sensitive attribute information.

#### 2.2.1 Sensitive attribute approximation

The most studied approach uses proxies for the sensitive attribute based on other features in the training data. There are two general strategies for approximating the sensitive attribute—inference using proxies and inference using correlates.

The first group of methods uses proxies to directly infer the sensitive attribute (D'Amour et al., 2020; Romanov et al., 2019; Zhang, 2018; Zhao et al., 2022; Grari et al., 2021). This method is commonly used to measure discrimination in real-world applications such as credit approval and health insurance pricing (Adjaye-Gbewonyo et al., 2014; Bureau, 2014). BISG (Bayesian Improved Surname Geocoding) developed by RAND Corporation is a popular tool to infer race. It uses Bayes Theorem and race distribution by surname and location from the Census Bureau to predict the race of individuals (Elliott et al., 2009). This tool was used by the Consumer Financial Protection Bureau in 2013 in a lawsuit against Ally Financial to identify racial discrimination in their lending decisions (Andriotis and Ensign, 2015). However, this method has limitations and its own biases. For example, BISG performs poorly on individuals who change their surname after interracial marriage (Argyle and Barber, 2024). This uncertainty in the sensitive attribute inference can negatively affect fairness measurements Although BISG is a fairly accurate method to infer gender, Chen et al. (2019) show that BISG tends to overestimate fairness and they provide a theoretical analysis of the amount of bias in the fairness estimates using the inferred sensitive attribute. Awasthi et al. (2021) further study this problem and provide more empirical analysis on fairness estimation using inferred sensitive attribute. They build multiple sensitive attribute inference models using random forest, logistic regression, SVM and a single hidden layer neural network model. All the models have a similar accuracy (ranging from 82% to 85%) of inferring the sensitive attribute. The authors then use the inferred sensitive attribute to measure the fairness and show that with very similar overall accuracy, different misclassification distributions can negatively affect the ability to measure the fairness. Our work differs from these previous works since our focus is not on identifying the best approach for inference, but rather to understand whether or not across different data sets fairness will improve (1) when there are different levels of inference accuracy of the sensitive attribute, and (2) when using different bias mitigation algorithms.

The second group of methods uses features that are highly correlated to the sensitive attribute to improve fairness (Gupta et al., 2018; Romanov et al., 2019; Zhao et al., 2021). Gupta et al. (2018) propose proxy fairness, where they begin by defining a set of proxies that are close to the sensitive attribute. Closeness can be defined using different metrics such as correlation, cosine similarity and earth mover's distance. They then use the set of proxies as the sensitive attribute to improve fairness. Zhao et al. (2021) proposes a bias mitigation algorithm that minimizes the correlation between the output and features that are highly correlated to the sensitive attribute, thereby minimizing the correlation between the output and the actual sensitive attribute. Romanov et al. (2019) propose a similar approach for occupation classification that reduces the correlation between the predicted value (occupation) and word embedding of an individual's name. Their experimental results show that this method can be used to reduce race and gender bias.

#### 2.2.2 Privacy-preserving

The second approach considers privacy-preserving methods to avoid directly using sensitive attributes (Hu et al., 2021; Kilbertus et al., 2018). For example, Hu et al. (2021) propose a distributed, privacy-preserving fair learning framework that uses multiple local agents, each holding different sensitive demographic data. During the training process each agent learns a fair local dictionary and sends it to the modeler. The modeler then learns a fair model based on an aggregated dictionary. Kilbertus et al. (2018) use the same idea but instead of using local agents, they use multiparty computation that allows calculations and aggregations from each party without revealing the input. However, this approach is hard to achieve in practice as it needs all users to be online at the same time. This scenario differs from ours since we assume that the sensitive attribute is not available to anyone.

#### 2.2.3 Distributionally robust optimization

The third approach uses adversarial learning and a distributionally robust optimization (DRO) framework (Pezeshki et al., 2021; Sohoni et al., 2020; Kim et al., 2019; Hashimoto et al., 2018; Lahoti et al., 2020). This approach was originally designed to improve robustness and generalization of machine learning models by minimizing the worst case loss across all worst case distributions. In machines learning fairness, marginalized (unprivileged) groups often have the worst model performance (highest loss) and researchers use the idea of DRO to improve model performance on the unprivileged groups to improve overall fairness. We highlight two examples of this general approach here. Lahoti et al. (2020) propose adversarially reweighted learning. This method hypothesizes that the sensitive attribute is correlated with non-protected features and task labels. After training a machine learning model, they use the model to identify observations with high errors (losses) and label these observations as the unprivileged group and then using existing bias mitigation algorithms to improve the model performance on the unprivileged group. Kim et al. (2019) propose multi-accuracy auditing and a post-processing method to improve model accuracy across identifiable subgroups. Their approach uses a small set of labeled data with sensitive attribute information for auditing and identifies the groups with high errors. They then post-process the model by changing their prediction labels to reduce errors. This approach focuses more on model robustness and does not require any information about the sensitive attribute. It uses the correlation between the training data and the class label, making the assumption that observations with high prediction errors are from disadvantaged groups, i.e. the classifier performs poorly on observations from minority groups, and that improving model performance from the worst performing groups will improve overall fairness.

Our work focuses on using sensitive attribute approximation. Our goal is to study how uncertainities in both accuracy and misclassification distribution in the approximated sensitive attribute negatively affects the performance of existing popular bias mitigation algorithms with respect to fairness. We study the sensitivity of each bias mitigation algorithm to errors in the inferred sensitive attribute. This type of comparative analysis is important to better understand the limitations of different bias mitigation strategies when the sensitive attribute is not available.

### 2.3 Impacts of inferred sensitive attribute

There are some examples of exploring the impact of an inferred sensitive attribute on bias mitigation investigate how uncertainty and errors in demographic inference impact fairness in ranking algorithms. They use race and gender as sensitive attributes and find that researchers should not use inferred demographic data as input unless the inference results have very high accuracy. Awasthi et al. (2020) study how imperfect group information affects the performance of post-processing bias mitigation algorithms. Imperfect group information occurs when we do not have the sensitive attribute information, but we have proxies that are highly correlated to sensitive attribute that we can use to obtain knowledge about the sensitive attribute. The knowledge about the sensitive attribute is not perfectly accurate and thus it is referred to as imperfect group information. Our work differs from these since we focus on a broader set of bias mitigation algorithms that can be used for machine learning classification tasks more generally, not just on a ranking task or specific bias mitigation algorithms. We also conduct a more detailed case study using social media data that incorporates demographic inference algorithms specifically designed for social media.

Wang and Singh (2023) conduct a smaller scale analysis on text classification tasks using sensitive attribute inference models with different levels of accuracy. They present two case studies using textual social media data and use multiple existing gender inference models to infer the sensitive attribute and use the inferred sensitive attribute to apply bias mitigation algorithms to improve fairness. They show that this approach is effective when the accuracy of the inferred sensitive attribute is reasonable (ranging from 0.76 to 0.84). While their work considers the problem of the inferred sensitive attribute, it does not systematically study different levels of accuracy through simulation and does not systematically compare the performance of a wide range of bias mitigation strategies (pre-processing, in-process, and post-processing strategies).

## 3 Problem statement

This section begins with definitions and notation (Section 3.1). We then present the *missing sensitive attribute problem* in Section 3.2.

### 3.1 Definitions and notation

Let *X* = {*x*_1_, *x*_2_, ⋯ , *x*_*n*_} be a set of non-sensitive features used to train a prediction model containing *n* observations. Let *S* = {*s*_1_, *s*_2_, ⋯ , *s*_*n*_} be the binary sensitive attribute, where *s*_*i*_ is the sensitive attribute value for the *i*^*th*^ observation and *S* ∉ *X*. For the *i*^*th*^ observation, we say *s*_*i*_ = 0 if the observation is in the unprivileged group and *s*_*i*_ = 1 if the observation is in the privileged group. *Y* = {*y*_1_, *y*_2_, ⋯ , *y*_*n*_} is the binary label that the classifier wants to predict, where *y*_*i*_ = + if it is a positive outcome for the *i*^*th*^ observation, e.g., getting approved for a loan, and *y*_*i*_ = − if it is an negative outcome. Let *M* be a binary classifier that tries to predict outcome *Y*. Let Ŷ = {ŷ_1_, ŷ_2_, ⋯ , ŷ_*n*_} represent the predicted outcome. A standard classification task can be summarized as *M*(*X*) = Ŷ, where we train a classifier *M* on a set of features *X* and obtain a set of predictions Ŷ. From an accuracy perspective, we want Ŷ and *Y* to be as close as possible. From a fairness perspective, we want the classifier to perform equally well across the privileged and unprivileged groups in *S*.

### 3.2 Problem formation

Unfortunately, we do not always have access to *S*. While there are a number of concerns this raises, we are interested in understanding the impact on the fairness of different bias mitigation strategies when this sensitive attribute is *inferred*. This is important to determine since it will be difficult to improve the public perception of the trustworthiness of black-box AI systems without understanding the relationship between bias mitigation strategies, inferred sensitive attributes, and fairness.

**Figure 1:**
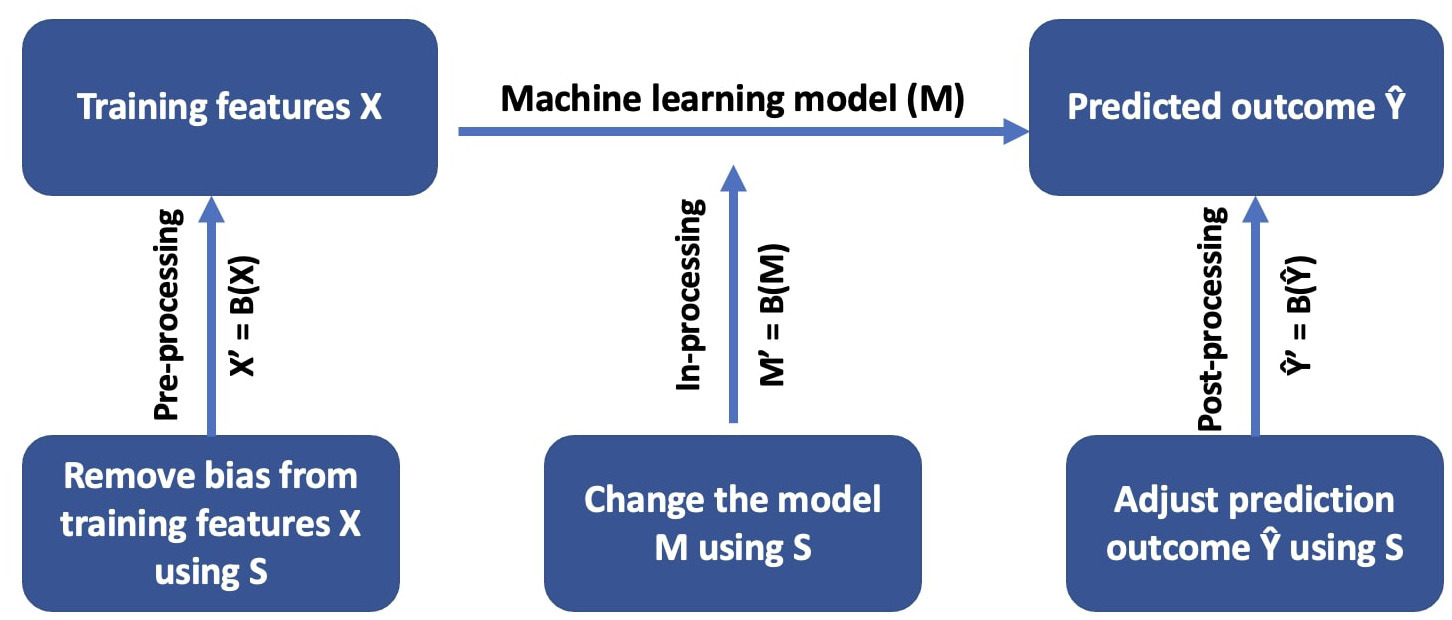
Bias mitigation mechanisms in different parts of the machine learning pipeline.

Figure 1 shows bias mitigation mechanisms for different parts of the machine learning pipeline. A typical machine learning pipeline contains a set of training features (*X*), a machine learning model (*M*), and prediction output (Ŷ). A bias mitigation mechanism *B* can be characterized by where it is applied within the pipeline, i.e., a pre-processing mechanism, an in-processing mechanism, or a post-processing mechanism. The goal of a pre-processing bias mitigation mechanism is to remove bias from training features *X* using information about sensitive attribute *S*. The result of a pre-processing bias mitigation mechanism is a new set of training features *X*′ that contain less bias, where *X*′ = *B*(*X*). In-processing bias mitigation mechanisms change the machine learning model *M* by using the information about the sensitive attribute *S*. One approach is to add a fairness constraint to the machine learning model. The result of an in-processing bias mitigation mechanism is a new model *M*′, where *M*′ = *B*(*M*), that is more fair than the original model *M*. Post-processing bias mitigation mechanisms improve fairness by changing the prediction outcome Ŷ based on sensitive attribute *S*. The result of a post-processing bias mitigation mechanism is a more fair prediction outcome Ŷ′, where Ŷ′ = *B*(Ŷ).

More formally, given a set of training features *X* and ground truth sensitive attribute *S*, we apply the bias mitigation method *B* and train a classifier to output a set of outcomes Ŷ or Ŷ′ depending upon where *B* is applied. We then evaluate the fairness of the classifier using sensitive attribute *S*. Without *S*, we cannot determine the effectiveness of the bias mitigation method nor use classic fairness measures to assess the fairness of the classifier. We refer to this problem as the *missing sensitive attribute problem*.

One strategy for measuring fairness when the sensitive attribute *S* is missing is to infer the sensitive attribute values (*S*′) and apply bias mitigation methods *B* using the inferred sensitive attribute *S*′. Let *Z* be a set of features that are used to infer the sensitive attribute *S* and *M*^*S*^ be the sensitive attribute inference model. Then *M*^*S*^(*Z*) = *S*′. The accuracy of the inferred sensitive attribute may affect the effectiveness of different bias mitigation methods. If the inference is very accurate (*S* ≈ *S*′), we expect different bias mitigation methods to have similar performance to that of using the ground truth sensitive attribute to assess fairness. However, for an inferred sensitive attribute with a moderate (and acceptable) accuracy, different bias mitigation methods may vary in performance, where some methods are more robust to the noisy sensitive attribute *S*′ and others are less robust. In this work, we explore when the accuracy of *S*′ negatively affects the performance of different bias mitigation methods and when it does not.

## 4 Methodology for determining the inferred sensitive attribute

**Figure 2:**
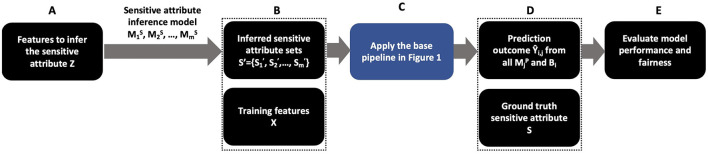
Overall design measuring the impact of uncertain sensitive attribute inference on bias mitigation algorithms.

Figure 2 shows the overall design for measuring how the inferred sensitive attribute impacts the performance of various bias mitigation algorithms. We use a set of features (*Z*) that is a subset of training features *X* or auxiliary features to infer the sensitive attribute depending on the prediction task. For example, in our case study in Section 6.3, *Z* ⊂ *X*. In BISG, *Z* ={surname, address} where surname and address are often not included in the training features to predict Ŷ. Using *Z*, we apply the sensitive attribute inference models $M_{j}^{S}$ for *j* = {1, 2, ⋯ , *m*} to get a set of inferred sensitive attribute predictions *S*′ (step A and B). These inferred values are used to attempt to reduce bias using *B*. *S*′ and *X* are then inputs into the base pipeline (step C with details in Figure 1). For each bias mitigation algorithm and inferred sensitive attribute, we have a prediction outcome Ŷ_*i,j*_ (step D). Finally, we evaluate the model performance and fairness using the ground truth sensitive attribute *S* and prediction outcome Ŷ_*i,j*_ (step E).

To understand how the accuracy of the inferred sensitive attribute impacts bias mitigation algorithms, we propose two strategies for obtaining the inferred sensitive attribute: (1) simulate sensitive attribute values with different error levels and misclassification distributions, and (2) use multiple existing sensitive attribute inference models. Using simulation allows us to systematically control the error rate. Using multiple existing sensitive attribute inference models allows us to evaluate the actual misclassification distribution of sensitive attribute inference models. The remainder of this section describes the steps associated with each of them.

### 4.1 Inferred sensitive attribute using simulation

Using two benchmark datasets for fairness, COMPAS and Credit card clients data, we obtain the inferred sensitive attribute through simulation. We choose the simulation approach on these data sets because (1) we want to systematically understand the robustness of different bias mitigation strategies to different levels of error in the inferred sensitive attribute, (2) both data sets are well studied, and (3) all the bias mitigation algorithms presented in this study have been shown to be effective on for improving fairness on at least one of the data sets.

Our simulation generates sensitive attribute values, meaning that we skip step A in Figure 2. We simulate inference results for the sensitive attribute $S_{j}^{\prime }$, each having a different level of accuracy: $S_{j}^{\prime }$, where *j* = 1, 2, ⋯ , *m*, and *m* represents the number of different sensitive attribute inference results we want to generate. We then use these simulated values for the sensitive attribute to assess the quality of each bias mitigation strategy in *B*. For example, suppose our simulated sensitive attribute is gender. We use the known gender values as the ground truth and generate (through simulation) the gender labels with different levels of accuracy. Then we test different bias mitigation strategies using these different sets of gender values. Algorithm 1 describes our approach more formally.

**Algorithm 1:**
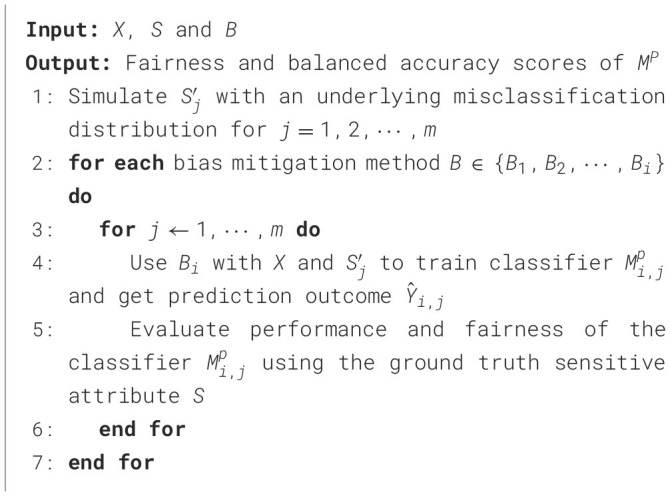
Methodology with inferred sensitive attribute results from simulation.

### 4.2 Inferred sensitive attribute from neural models

Although the simulation approach can simulate different levels of accuracy and a complete misclassification distribution of the inferred sensitive attribute, they are still simulated results. To compliment our simulation results, we explore uncertainties related to the misclassification distributions based on using real models. The Wikidata (see description in next section) allows us to test inferred sensitive attributes using multiple existing demographic inference models on social media data. The sensitive attribute is gender and we use three different state of the art gender inference models (*M*^*S*^), each having a different misclassification distribution, to generate *S*′. Algorithm 2 presents this approach.

**Algorithm 2:**
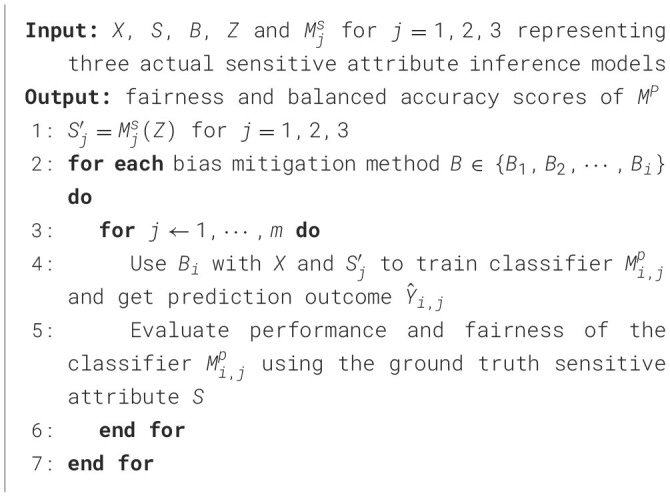
Methodology with inferred sensitive attribute results from actual models.

## 5 Experiment setup

In this section, we will discuss the three data sets we use (Section 5.1), the bias mitigation methods we test (Section 5.2), and the evaluation metrics for measuring fairness and accuracy for our classification tasks (Section 5.3).

### 5.1 Data sets

#### 5.1.1 COMPAS recidivism data

The COMPAS recidivism risk data set (Jeff et al., 2016) contains 7,214 observations with 14 features. The goal is to predict whether an individual recidivated. If an individual did not recidivate, we label that as a positive outcome. If an individual recidivated, we label that as a negative outcome. Race is the sensitive attribute, with black as the unprivileged group and non-black as the privileged group.

#### 5.1.2 Credit card clients data

The credit card clients data set (Dua and Graff, 2017) contains 30,000 observations with 22 features. We want to predict if a client will default on the credit card payment, with not defaulting as a positive outcome and defaulting as a negative outcome. Gender is the sensitive attribute, with female as the unprivileged group and male as the privileged group.

#### 5.1.3 Wikidata

We use an existing Wiki data set containing a set of politicians who have shared their demographic information and their Twitter handles (Liu et al., 2021). This data set contains 736 observations. We use the Twitter API to collect the posts shared by the politicians. The prediction task is to use the Twitter post content to determine the political party of the politician. The party is either Democratic or Republican with Republican as the minority group.^3^ Gender is the sensitive attribute with male as the privileged group and female as the unprivileged group. For the classic machine learning models, we construct N-gram features from tweets as the training feature for this task. In the deep learning model, we convert the text into embeddings and use the embedding vectors in a LSTM neural network with an attention mechanism. We will present details about the model architecture in Section 6.3.

### 5.2 Bias mitigation methods

We explore the negative effect of uncertainty in *S*′ (the inferred sensitive attribute) when using different bias mitigation methods (*B*) for predicting *Y*. We consider six bias mitigation methods introduced in Section 2.1: three pre-processing mechanisms: Resampling (RS) (Wang and Singh, 2021), Reweighting (RW) (Kamiran and Calders, 2012) and Disparate Impact Remover (DIR) (Feldman et al., 2015), two in-processing mechanisms: Adversarial Debiasing (AD) (Zhang et al., 2018) and Exponentiated Gradient Reduction (EGR) (Agarwal et al., 2018), and one for post-processing mechanism: Threshold Optimizer (TO) (Hardt et al., 2016).

### 5.3 Evaluation metric

#### 5.3.1 Fairness

In recent year, a number of approaches have been proposed for measuring fairness of machine learning models, including disparate impact (p%-rule) (Feldman et al., 2015; Zafar et al., 2017), demographic parity (Calders and Verwer, 2010; Dwork et al., 2012), equalized odds (Hardt et al., 2016), and equal opportunity (Hardt et al., 2016). Among all fairness metrics, disparate impact is the closest to the legal definition of fairness and is often used in anti-discrimination law to quantify fairness and discrimination (Barocas and Selbst, 2016). For these reasons, we use disparate impact (p%-rule). It is defined as:


$$\text{min}\left(\frac{P\left(Ŷ=+|S=1\right)}{P\left(Ŷ=+|S=0\right)},\frac{P\left(Ŷ=+|S=0\right)}{P\left(Ŷ=+|S=1\right)}\right)$$

Generally, if the disparate impact value is greater than 80%, or 0.8, the classifier is considered to be non-discriminatory (Biddle, 2017).

#### 5.3.2 Outcome prediction model performance

We use balanced accuracy score (Brodersen et al., 2010) to evaluate performance of the inference models *M*^*s*^ and machine learning models *M*^*p*^. It is defined as:


$$\text{balanced-accuracy}=\frac{1}{2}\left(\frac{TP}{TP+FN}+\frac{TN}{TN+FP}\right)$$

where *TP* represents true positive, *TN* represents true negative, *FP* represents false positive, and *FN* represents false negative. Compared to the accuracy and F1 score, the balanced accuracy score avoids inflated performance estimates on imbalanced data sets, especially imbalanced data sets that are more concerned with the negative labels.

## 6 Results

This section begins by presenting the machine learning models we use for predicting *Y* for each of the three data sets (Section 6.1). We then present the results using the simulated sensitive attributes for the COMPAS and credit card client data (Section 6.2). This is followed by the social media case study using Wikidata and an inferred sensitive attribute from different machine learning models (Section 6.3). Finally, we present a sensitivity analysis of the different bias mitigation methods (Section 6.4).

### 6.1 Selecting a machine learning model

Our main task is to predict *Y* using the attributes in *X* and maintain high accuracy and high fairness. In this section, we compare a variety of machine learning models from classic ones to a deep neural network. More specifically, we use the following four learning models for all dataset to determine *M*^*p*^: random forest, logistic regression, support vector machine (SVM) with a Gaussian kernel, and a multilayer perceptron neural network for the COMPAS and the credit card client data. There are many possible parameters in a neural network model. We tested four different settings on neural models with 2 hidden layers and 3 hidden layers and each layer has 50 and 100 hidden nodes to perform a sensitivity analysis. All the settings had similar results. Our results presented show the best performing models. In the COMPAS data, the best neural model has 2 hidden layers and 50 hidden nodes in each layer and in the credit card client data, the best model has 2 hidden layers and 100 hidden nodes in each layer.

For the text-based Wikidata, we use a deep neural network with an attention mechanism instead of the neural model used for the other two data sets. Specifically, we use the model architecture presented by Wang and Singh (2023). This architecture first converts tweets and user biographies into embeddings using the pretrained uncased BERT-Base model (Reimers and Gurevych, 2019). It then inputs embedding vectors into a Long Short Term Memory (LSTM) layer. Finally, it adds account information features together into an attention layer that combines all the information and selects the part that is more informative into a fully connected Multi-Layer Perceptron neural network with one hidden layer and 50 hidden nodes. In all the neural models, there are common hyperparameters such as learning rate and number of epochs. To determine the hyperparameters, we use the loss on the validation set to adjust the learning rate and use early stopping to determine the number of epochs. The learning rate is 0.001 for the COMPAS data set, 0.0025 for the credit card client data set and 0.0025 for the Wikidata data set. The number of epochs with early stopping are as follows: 68 for COMPAS data, 52 for card client data, and 93 for Wikidata. All other hyperparameters such as parameters in the PyTorch optimizers and the cross entropy loss function are set to be the default values in the PyTorch package.

Our evaluation uses an 80/20 split and uses all the features in *X*, not including *S*. We use 80/20 train test split because we want to be consistent across our simulation study. We note that we did conduct 5-fold cross validation and the difference in results is negligible to those we present here. We call this model the *standard model* since we use all features in *X* to train each classifier without incorporating any bias mitigation algorithms.

Table 1 shows the balanced accuracy score on the test data set for each of the learning models. On all three data sets, the random forest classifier has the best performance, ranging from two to six percent better than the other classifiers. For the experiments that follow, we use a random forest classifier for the basic classification task since it had the best performance on all the data sets. For comparison, we present our results on other prediction models in Appendices A, B.

**Table 1:** Balanced accuracy score of the standard models using all the training features *X*.

**Machine learning model**	**COMPAS**	**Credit card client**	**Wikidata**
Random forest	0.693	0.758	0.84
Logistic regression	0.672	0.69	0.822
SVM	0.679	0.703	0.787
Neural network	0.682	0.751	0.833

### 6.2 Simulation study

Recall that the goal of this simulation study is to understand how the accuracy and misclassification distributions of the inferred sensitive attribute *S*′ impacts the performance of various bias mitigation algorithms applied at different points in the machine learning life cycle. We consider 11 different error levels for the sensitive attribute: $S_{1}^{\prime },S_{2}^{\prime },\cdots \;,S_{11}^{\prime }$, where each inferred result has a different accuracy. $S_{1}^{\prime }$ has the lowest accuracy (0%) and $S_{11}^{\prime }$ has the highest accuracy (100%).

In order to simulate each sensitive attribute value, we use the following procedure. Beginning with the ground truth sensitive attribute (accuracy = 100%), we select some observations in the data set and change the value of the sensitive attribute. This means that the simulated results will deteriorate as more of these values are flipped. For example to simulate an inference result with 0.8 accuracy, we randomly select 20% of the observations from the sample and change the sensitive attribute value (e.g., male to female or female to male). In a sense, our simulator acts as a hypothetical machine learning model for *M*^*s*^ that uses features *Z* to simulate different misclassification distributions for *S*. Different misclassification distributions are able to represent bias in inferred sensitive attribute. Recall that in Section 2.2.1, we mentioned that BISG (Bayesian Improved Surname Geocoding) is a popular tool used by lenders to infer each applicant's race using the last name and location (Adjaye-Gbewonyo et al., 2014; Bureau, 2014) even though this method is biased against inter-racial married females. Using different misclassification distributions allows us to reflect such bias in sensitive attribute inference.

To capture some variability that can occur in the real sensitive attribute inference models, we tested inferred results with three different misclassification distributions: a random misclassification, a stratified misclassification in which the privileged group has a higher accuracy, and a stratified misclassification in which the unprivileged group has a higher accuracy. Table 2 shows examples of these misclassification distributions. All of them have the same overall balanced accuracy score. In the random misclassification experiments (the leftmost column), both the privileged and unprivileged groups have the same accuracy. In the middle column, the unprivileged group has a higher inference accuracy and in the rightmost column, the privileged group has a higher inference accuracy.^4^

**Table 2:** Misclassification distributions of simulated sensitive attribute results.

**Inferred sensitive attribute accuracy**	**Random misclassification**	**Higher accuracy on unprivileged group**	**Higher accuracy on privileged group**
Privileged group accuracy	0.8	0.72	0.88
Unprivileged group accuracy	0.8	0.88	0.72
Overall balanced accuracy	0.8	0.8	0.8

We split each *S*′ (having different error rates) into a training set and a test set. We use the inferred sensitive attribute (*S*′) in the training set, apply the bias mitigation method (*B*) we are testing, and train a classifier to predict the label (*Y*). As mentioned in the previous subsection, we use the Random Forest classifier (see Table 1 for classifier comparison) to assess the sensitivity of the bias mitigation methods for different misclassification distributions and accuracy levels of *S*′.

For the validation, if the bias mitigation method requires a sensitive attribute from the test set (this is the case for the threshold optimizer and disparate impact remover), we use the inferred sensitive attribute in the test set to perform the bias mitigation. We then evaluate the performance and fairness of the classifier using the ground truth sensitive attribute (*S*) and ground truth labels (*Y*).

In this paper, We use Python 3.8 on a Mac with M2 chip and 16 GB memory. Each experiment including inferring the sensitive attribute and apply the bias mitigation algorithms takes less than 2 min. Pre-processing and post-processing algorithms are the most efficient, with each experiment taking less than 30 seconds. In-processing algorithms are the least efficient, with Adversal Debiasing (AD) taking approximately 2 min.

#### 6.2.1 COMPAS experiment

**Figure 3:**
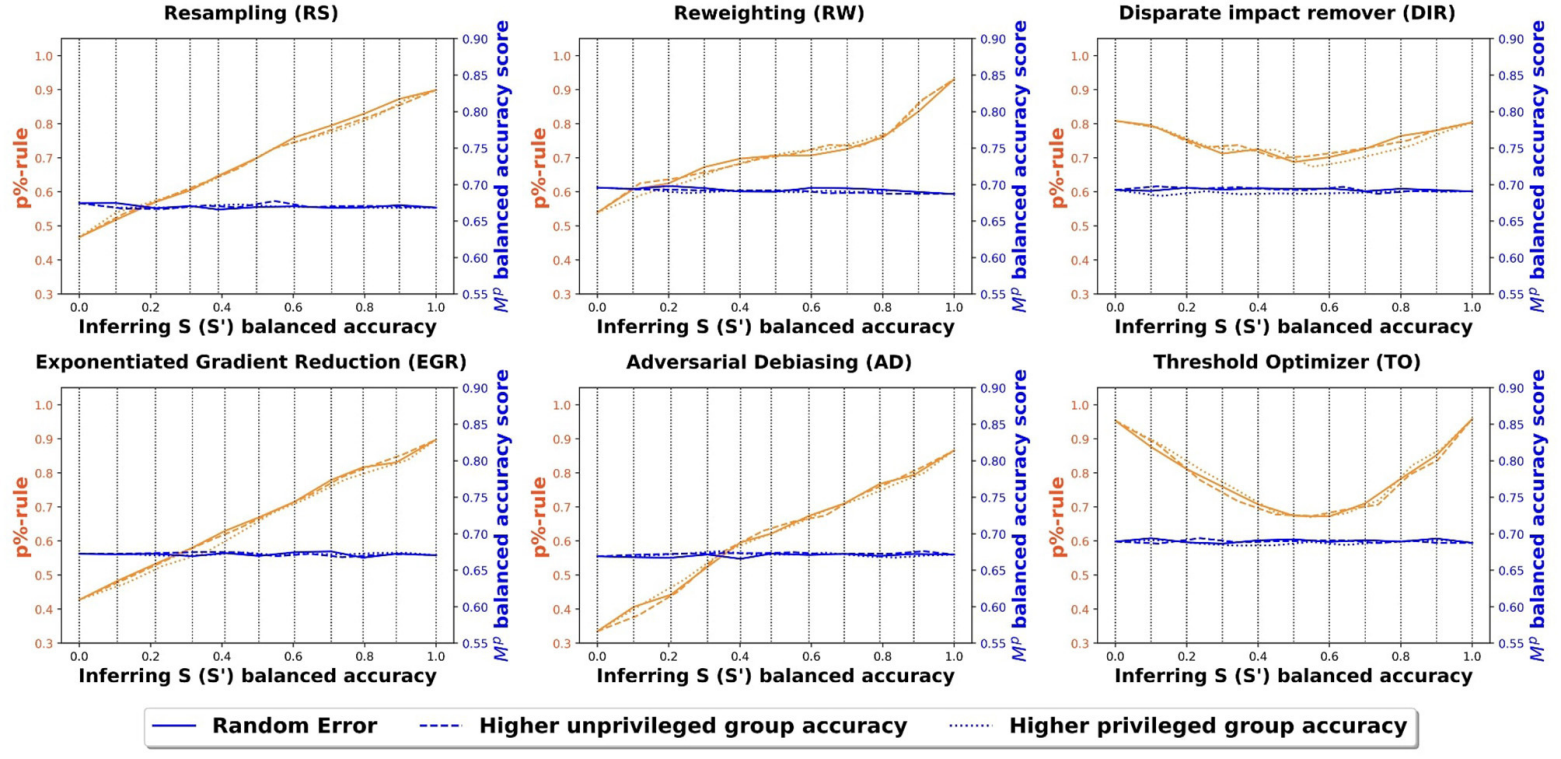
Balanced accuracy and fairness scores of the outcome prediction models using COMPAS data.

Figure 3 shows the fairness and balanced accuracy of the machine learning models (*M*^*p*^) across different accuracies of the inferred sensitive attribute for different bias mitigation strategies. Each subfigure corresponds to a specific bias mitigation strategy. The x-axis shows the different levels of balanced accuracy for inferring the sensitive attribute ($M_{1}^{s},\cdots \;,M_{11}^{s}$). The left y-axis shows the fairness score (p%-rule) for the outcome prediction model *M*^*p*^ and the right y-axis shows the balanced accuracy score for the outcome prediction model *M*^*p*^. Each vertical dashed line highlights the balanced accuracy and the fairness for *M*^*s*^ at a specific accuracy level. The figure shows the balanced accuracy using *M*^*p*^ to predict *Y* (blue lines), and fairness scores (p%-rule) for different sensitive attribute models *M*^*s*^ (yellow lines). The three lines represent different misclassification distributions of *M*^*s*^ with the solid lines representing random misclassification, dashed lines for higher unprivileged group inference accuracy, and dotted lines for higher privileged group inference accuracy. For visual clarity, we choose not to show the error bars. However, we note that across all figures, the standard errors are within 0.025.

In the COMPASS data set, across the three misclassification distributions (the orange lines), the overall trends are consistent and the results are very similar to each other. The model performance (balanced accuracy) across all bias mitigation methods remains fairly constant. This is not surprising since all mitigation methods aim to achieve high accuracy while improving fairness. We do see trade-offs between fairness and accuracy, but the trade-offs are very small. For all the bias mitigation methods, except the threshold optimizer and the disparate impact remover, the fairness score increases substantially as the sensitive attribute inference model becomes more accurate, but the prediction model accuracy remains fairly constant. While not surprising, this result is an indication that the bias mitigation methods are sensitive to the balanced accuracy of the inferred sensitive attribute, and that some bias mitigation methods are more sensitive than others.

For the threshold optimizer and the disparate impact remover, there is a “V” shaped fairness score. As we mentioned earlier, both the threshold optimizer and the disparate impact remover require sensitive attribute information in the test set. Recall, that for this experiment, we used the inferred sensitive attribute values that are part of the test set. Because our sensitive attribute is binary, a perfect sensitive attribute inference model is equivalent to an inference model with 0% accuracy since all the prediction results for *S*′ are set to the opposite class. For example, in a sensitive attribute inference model that has 0% accuracy, all the female examples are predicted as male and all the male examples are predicted as female. Because the threshold optimizer sets a new probability threshold to ensure equal outcomes, the fairness will be a mirror image of the flipped sensitive attribute accuracy. This results in a V shape. Clearly, we want to focus on sensitive attributes that are more than 50% accurate. But we show the full range of sensitive attribute accuracies for completeness.

In the bias mitigation methods that do not use the sensitive attribute from the test set, the fairness score decreases significantly when the accuracy of sensitive attribute inference model is low (<0.5). When the accuracy of the inferred sensitive attribute is low, the bias mitigation method benefits the unprivileged group. For example, with a very bad inference model, the reweighting method tends to put higher weights on over-represented groups and lower weights on under-represented groups. While the fairness scores are highest for the threshold optimizer when the sensitive attribute accuracy is high (greater than 90%), all the methods perform similarly when the sensitive attribute accuracy is moderate (70%–80%).

#### 6.2.2 Credit card clients experiment

**Figure 4:**
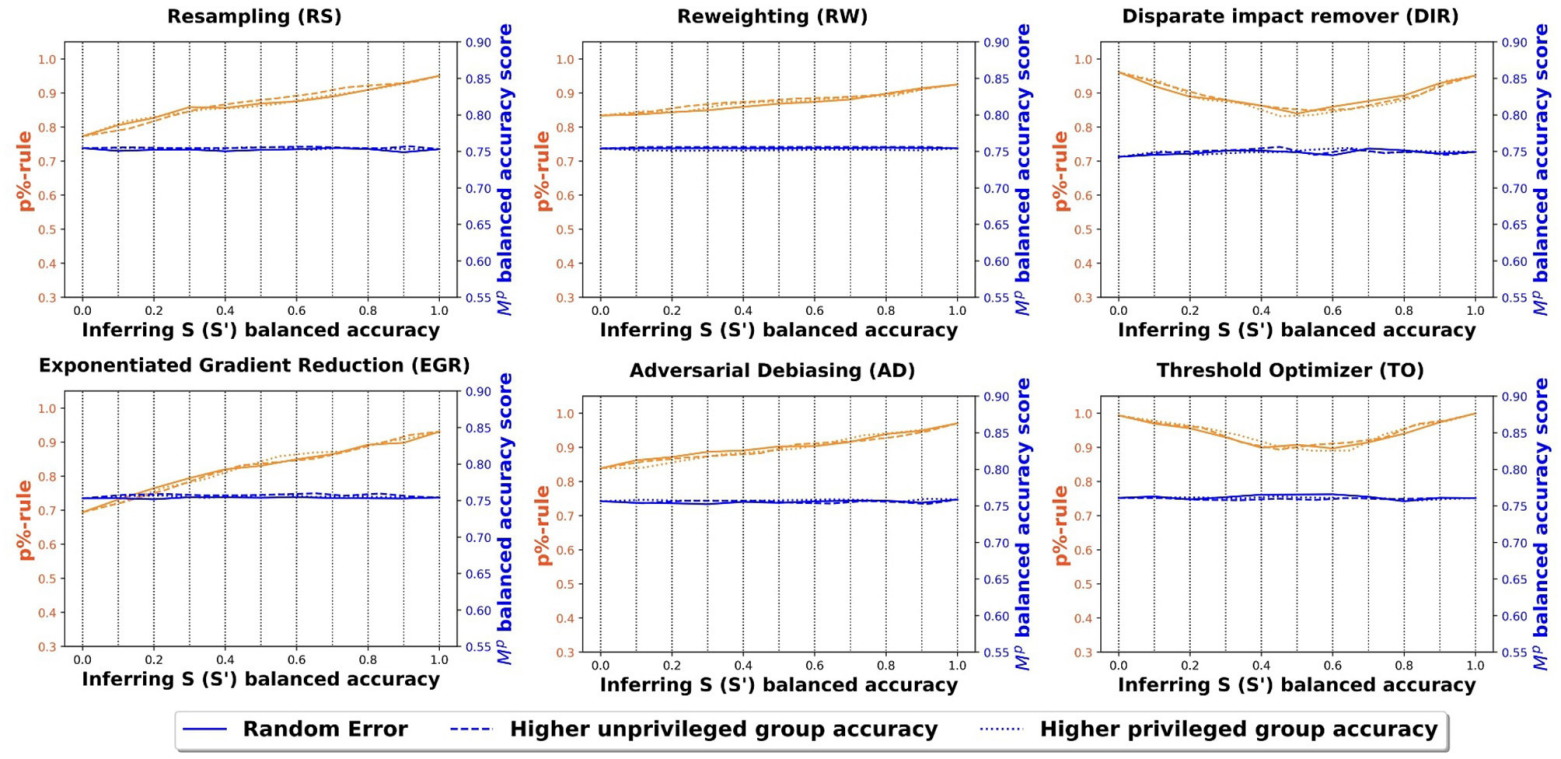
Balanced accuracy and fairness scores of the outcome prediction models using credit card clients data.

Figure 4 shows the fairness and balanced accuracy of the prediction model for the credit card client data set. The information and the axes of the subfigures are the same as Figure 3. Similar to the COMPAS data set, all three inference misclassification distributions have very similar fairness and accuracy curves. In other words, the distribution of the misclassified values in *S*′ does not impact the overall fairness or balanced accuracy of Ŷ. Once again, threshold optimizer and disparate impact remover have “V” shaped fairness scores and all the other methods have a positive relationship between the fairness scores and the accuracy of *S*′. Across all the bias mitigation methods, the balanced accuracy scores of the outcome prediction models remain fairly constant. Compared to the COMPAS data set, the fairness scores in the credit card clients data set are better because the credit card clients data set has less bias in the original data set. Once again, the fairness scores are highest for the threshold optimizer when the sensitive attribute accuracy is high (greater than 90%), and similar across all methods when the sensitive attribute accuracy is moderate (70%–80%).

### 6.3 Case study with Wikidata

Recall that in Section 4.2, we mentioned that we use the case study and actual existing sensitive attribute inference models to explore uncertainties in the misclassification distribution of real models. In this case study, we use three different state of the art gender inference models (*M*^*s*^) to generate *S*′ and see the impact of each of these inference models on the results of bias mitigation. All are neural models that use different language models and attention mechanisms to infer gender. One model uses tweets where the model maps each tweet into an embedding space using BERT and then each user is represented as the summation of all that user's mapped embeddings (Liu et al., 2021). The next model takes advantage of a hierarchical architecture that uses a GRU (Gated Recurrent Unit) with an attention layer to separately train the emoji component [using word embeddings and a CNN (Convolutional Neural Network)] and the text component (using BERT) of a Twitter post (Liu and Singh, 2021). The final model maps both tweets and images into an embedding space using CLIP (Contrastive Language-Image Pre-training), then uses GRU layers with an attention mechanism to process the embeddings and make predictions. This neural model has the following hyperparameters: batch size: 32, learning rate 0.0001, word embedding dimension: 50, sentence embedding dimension 768, filter window sizes: 2, 3, 4, filter number for each size: 256, emoji threshold length: 30 and maximum number of tweets per users: 200 (Liu and Singh, 2021).

Table 3 shows the balanced accuracy of *S*′ from the three sensitive attribute inference models *M*^*s*^. BERT emoji has the best overall balanced accuracy score (0.765). Recall that in Section 2.3, our case study in this work focuses more on misclassification distribution. When considering the privileged group accuracy compared to the unprivileged group, the privileged group has an inference accuracy that is 15% to 30% higher. In other words, all of these models have a reasonable overall accuracy, but perform better for the privileged group.

**Table 3:** Gender inference accuracy using existing demographic inference models.

**Inferred sensitive attribute accuracy**	**CLIP image**	**BERT emoji**	**BERT text**
Privileged group accuracy	0.832	0.855	0.878
Unprivileged group accuracy	0.679	0.675	0.567
Overall balanced accuracy	0.756	0.765	0.722

Table 4 shows the prediction model (*M*^*p*^) performance and fairness of the Wikidata using the inferred sensitive attribute values *S*′ from *M*^*s*^ and the ground truth (actual) sensitive attribute *S*, allowing us to easily compare the impact of the inferred sensitive attribute on the bias mitigation approach. Across the bias mitigation approaches, the balanced accuracy for all three sensitive attribute inference models (CLIP image, BERT emoji, BERT text) is similar to the balanced accuracy when using the ground truth sensitive attribute. In contrast, there are differences in the p%-rule across bias mitigation approaches. When using *S*′, exponential gradient reduction (EGR) is the best bias mitigation method (p%-rule = 0.733). It has the highest fairness score across all the different methods for computing *S*′, and is between 2% and 5% higher than the other bias mitigation methods. We also see a significant difference in p%-rule between *S* vs. *S*′. The best bias mitigation approach in terms of p%-rule when using *S* is threshold optimizer (p%-rule = 0.939). This means that there is a 20% difference in p%-rule when using *S* vs *S*′. Given this finding, we compare the fairness of *M*^*p*^ when bias mitigation is not used to the fairness of *M*^*p*^ when *S*′ is used with different bias mitigation methods to determine whether or not there is any improvement. Table 5 shows the p%-rule when *S* and no bias mitigation method is employed. For the Wikidata, the p%-rule is 0.52, 21% lower than when using *S*′ with EGR for bias mitigation. This means that while using bias mitigation strategies with *S* results in a better p%-rule score than using them with *S*′, even with different misclassification distributions, using a bias mitigation strategy with *S*′ is much better than not using any bias mitigation strategy at all.

**Table 4:** Balanced accuracy and fairness score of the outcome prediction model (using inferred sensitive attribute) on Wikidata.

**Bias mitigation algorithm**	**Balanced accuracy**				**p%-rule**			
	**CLIP image**	**BERT emoji**	**BERT text**	**Actual** *S*	**CLIP image**	**BERT emoji**	**BERT text**	**Actual**
RS	0.834	0.833	0.836	0.831	0.693	0.681	0.699	0.899
RW	0.838	0.837	0.836	0.835	0.718	0.695	0.709	0.908
DIR	0.831	0.832	0.834	0.832	0.672	0.681	0.683	0.843
AD	0.839	0.835	0.837	0.834	0.681	0.682	0.674	0.862
EGR	0.828	0.825	0.824	0.827	0.733	0.726	0.721	0.894
TO	0.837	0.839	0.834	0.831	0.713	0.704	0.708	0.939

**Table 5:** Fairness and performance in the baseline prediction model.

	**COMPAS**	**Credit card client**	**Wikidata**
Balanced accuracy score	0.693	0.758	0.84
p%-rule	0.668	0.861	0.52

### 6.4 Assessing the robustness of the bias mitigation methods

Given the differences in p%-rule in the Wikidata, we consider the scenario when the accuracies of the model used to infer the sensitive attribute are “average” (0.75 balanced accuracy) instead of “high” (0.90 balanced accuracy) and explore the performance of the different bias mitigation methods. We choose 0.75 balanced accuracy because in many real world machine learning applications, the prediction accuracy is mostly between 0.7 and 0.9. For example, machine learning models predicting various diseases such as heart disease, difference cancers, diabetes and stroke generally have accuracies between 0.7 and 0.9 (Uddin et al., 2019) and this accuracy is similar to doctor judgments (approximately 0.8) (Graber, 2013).^5^

**Figure 5:**
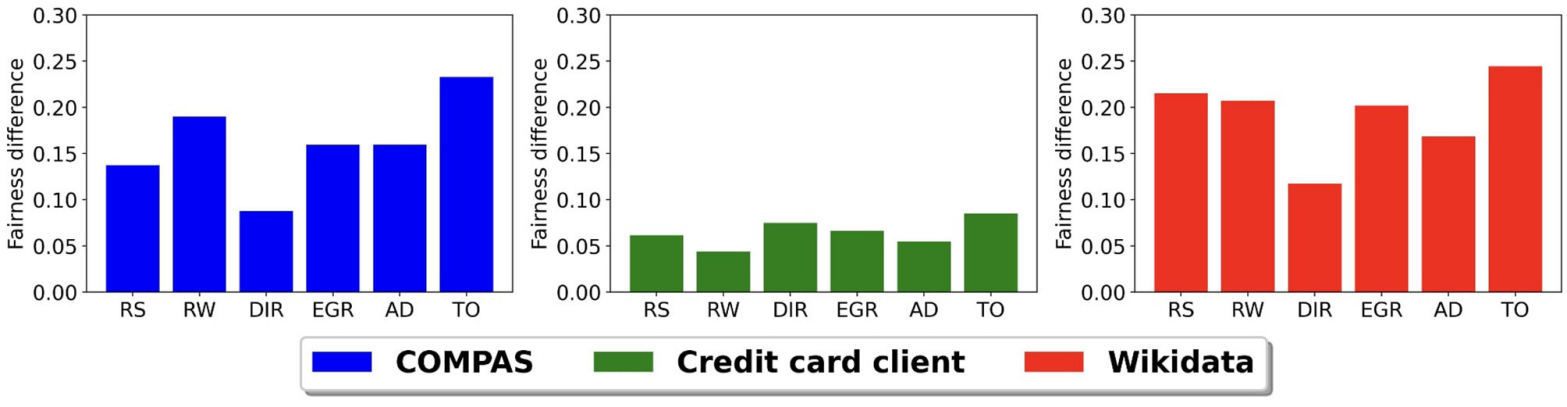
Prediction model fairness difference using the ground truth sensitive attribute *S* and the inferred sensitive attribute *S*′ with 0.75 balanced accuracy.

Figure 5 shows the difference in model fairness of *M*^*p*^ when using *S* and *S*′ with a balanced accuracy of 0.75 for our three data sets. For the COMPAS and credit card client data sets, we use the simulated sensitive attribute inference model results with random misclassification at 0.75 balanced accuracy. We chose random misclassification because the earlier simulation study showed that different misclassification distributions did not significantly affect the prediction accuracy and fairness. For the Wikidata, we use the CLIP image model to infer *S*′. The y-axis in Figure 5 shows the difference between the fairness scores of *M*^*p*^ using the ground truth sensitive attribute *S* and the inferred sensitive attribute *S*′ having a 0.75 balanced accuracy. The x-axis shows the different bias mitigation methods. We only present the fairness difference in the figure because the difference in balanced accuracy is very small, within 2% across all models and data sets. Given the findings in the previous subsection, it is no surprise that the fairness scores are the highest when using the ground truth sensitive attribute. The Wikidata and the COMPAS data set have the largest differences, while the credit card clients data set has the smallest difference. This is likely a result of the larger levels of bias in the original Wikidata and COMPAS data sets when compared to the credit card clients data set.

When comparing bias mitigation methods, we see that threshold optimizer (TO) and resampling (RS) are the most sensitive to uncertainties in *S*′ and disparate impact remover (DIR) is the least sensitive. The bias mitigation method sensitivity depends on the reliance of the method on the sensitive attribute. In the threshold optimizer and the resampling methods, they directly use the sensitive attribute values to improve fairness. For example, the resampling method uses sensitive attribute values to decide if a group is under-represented or over-represented and then performs resampling. On the other hand, the disparate impact remover method adjusts the earth mover's distance between feature distributions of the two sensitive groups. When compared to the threshold optimizer and the resampling methods, it uses the sensitive attribute more indirectly.

#### 6.4.1 Comparing the standard model fairness to fairness after using bias mitigation methods

We now compare the fairness of a model before and after using bias mitigation. Recal that we refer to a model that does not use any bias mitigation as a *standard model*. Table 5 shows the fairness and balanced accuracy scores of the best standard model for each data set. Among the three data sets, the Wikidata data set has the highest bias and the credit card client data set has the least bias. This is consistent with our earlier findings that the fairness scores in the credit card client data are higher than the COMPAS set, especially when the inference models have relatively poor performance. In particular, the slopes of the fairness curves in Figure 3 are steeper than the slopes in Figure 4 because in the original data set, the credit client data has less bias since the training features (*X*) are less correlated to the sensitive attribute (*S*). Consequently, in less biased data sets, bias mitigation algorithms do not need to modify the input data (pre-processing algorithms) or the model (in-processing algorithms) or the output (post-processing algorithms) as much to improve fairness. Thus, bias mitigation algorithms will be less sensitive to uncertain sensitive attributes.

**Figure 6:**
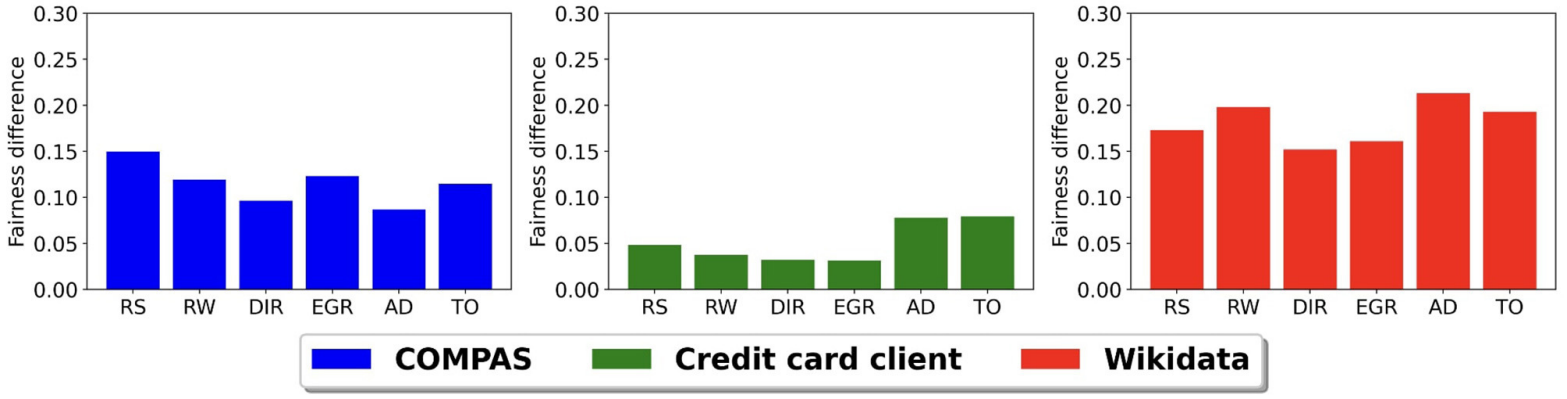
Prediction model fairness difference between baseline model and bias mitigation methods using inferred sensitive attribute with 0.75 balanced accuracy.

Figure 6 shows the difference between the fairness of the baseline model and the model after using each bias mitigation method with an inferred sensitive attribute having a balanced accuracy of 0.75. Again, we use the CLIP model to infer *S*′ in the Wikidata. Similar to Figure 5, we only present the fairness difference because accuracy scores change very little (within 2%) across all models and data sets. We see that all six bias mitigation methods have higher fairness scores than the standard model across all the data sets. The fairness difference in the Wikidata data set is the largest and again, the smallest is in credit clients data set. There is no one bias mitigation method that consistently has the highest difference.

An important takeaway from these last two experiments is that it is possible to improve the fairness of AI systems even when the ground truth sensitive attribute is not available when using an inferred sensitive attribute with reasonable accuracy (75%) with any of these different bias mitigation strategies. This is also the case when there is a bias in the accuracy of the sensitive attribute, i.e., the accuracy is not random.

## 7 Conclusion and future work

Given the increased reliance on black-box AI systems, it is imperative that we develop methods to ensure their fairness. In this study, we consider the scenario of needing to determine fairness when a ground truth sensitive attribute is not available to a researcher. We investigate the viability of using different bias mitigation strategies with an inferred sensitive ground truth attribute when this scenario occurs. We consider different bias mitigation strategies that are applied at different points in the machine learning life cycle and evaluate their effectiveness on three data sets. Our results show that different bias mitigation algorithms have different levels of sensitivity with respect to the accuracy of the inferred sensitive attribute. Across the six bias mitigation algorithms we tested, the threshold optimizer method and the resampling method were the most sensitive and the disparate impact remover method was the least sensitive. Ultimately, the sensitivity depended on the reliance of the bias mitigation algorithm on the sensitive attribute. The more reliant the strategy is on the sensitive attribute, the more important it is to have a high accuracy for the inferred sensitive attribute.

We also find that if we apply the bias mitigation algorithms using an inferred sensitive attribute with reasonable accuracy, the fairness scores are significantly higher than the best standard model and the balanced accuracy is similar to that of the standard model. In other words, bias mitigation across all stages of the life cycle always improves fairness if the accuracy of the inferred sensitive attribute is reasonable, even when the error within the inferred sensitive attribute is biased. This finding is critical for two reasons. First, it is an indication that bias mitigation at any stage in the life cycle can have a positive impact, even when the sensitive attribute is not available. Second, sometimes the inferred sensitive attribute may have its own bias, and our results show that some bias in the inferred sensitive attribute still leads to improved fairness results. Given these findings, we suggest that AI systems, in general, should develop procedures that incorporate bias mitigation prior to using the system even when the sensitive attribute needs to be inferred and the accuracy of the inference is moderate.

### 7.1 Possible implications

The missing sensitive attribute problem is very common in many real world data sets and applications. This work demonstrates that if a reliable method to infer the sensitive attribute is used, researchers and practitioners can improve fairness on data sets and applications. In other words, as a community, we can begin building models that infer different sensitive attributes and use these models to improve equity and fairness in real world systems. The research community has been developing different models for inferring a range of sensitive attributes (gender, age, location, political affilation, etc.) (Liu and Singh, 2023, 2021, 2024; Culotta et al., 2015). It is time to begin to use these models and methods for inferring demographics to improve fairness more broadly in AI systems that do not have sensitive attribute information, particularly black-box AI systems.

From a practical standpoint, when working with binary sensitive attributes, we recommend researchers only use an inferred sensitive attribute to improve model fairness if they have access to a binary sensitive inference model with an accuracy of at least 75%. Researchers can choose the bias mitigation algorithm based on the sensitive attribute inference accuracy. If the sensitive attribute inference accuracy is relatively low, around 75%, researchers can use bias mitigation strategies that are less dependent on that accuracy such as Disparate Impact Remover (DIR). If the accuracy is relatively high like above 90%, researchers can use any of the six bias mitigation strategies we tested, including ones that are more sensitive to inferred sensitive attribute accuracy [adversarial debiasing (AD) and Threshold Optimizer (TO)].

### 7.2 Future work

While there are many extensions for this work, we present three.

#### 7.2.1 Fairness of sparse, noisy data

In the three data sets we present in this work, the Twitter/X data set built using Wikidata has the largest bias. Unlike established fairness data sets, we often do not have ground truth sensitive attribute information for social media data sets, making it difficult for researchers designing algorithms in that arena to detect the bias and mitigate the bias. Given the complexities of the Twitter/X data compared to the other two data sets and the higher levels of bias when the sensitive attribute is inferred, future work should consider fairness for non-binary sensitive attributes and real world data sets that contain noisy, missing, and sparse text features.

#### 7.2.2 Multivariate sensitive attributes

Multivariate sensitive attributes are more complex and often require different frameworks. There are two types of multivariate sensitive attributes: single attribute and intersectional attributes. When there is only a single attribute, fairness metrics are similar to the binary sensitive attribute case. Bias mitigation algorithms for multivariate sensitive attributes are very different (Kang et al., 2022; Ma et al., 2021; Chen et al., 2024). To extend our work to multivariate sensitive attributes, future work needs to consider bias mitigation methods for multivariate sensitive attributes at different stages: pre-processing (Kamiran and Calders, 2012; Feldman et al., 2015; Chakraborty et al., 2021), in-processing (Tarzanagh et al., 2023; Shui et al., 2022; Chen et al., 2022; Peng et al., 2022), and post-processing (Hardt et al., 2016; Pleiss et al., 2017). We would expect that predicting a multivariate sensitive attribute would have worse performance than a binary one. If we want to use the same correction approach with multivariate sensitive attributes, we need to choose sensitive attributes with very few categories or apply binning to get reliable inferred sensitive attribute information. For example, inferring the exact age using social media data is very difficult. Researchers often bin the age into three or four bins and build models to infer age bins (Liu and Singh, 2021; Chen et al., 2015; Wang et al., 2019). In location inference there are different granularity in locations from the exact address to the state and country. Existing location inference models mostly focus on city and state level (Simanjuntak et al., 2022; Beigi and Liu, 2020; Cho et al., 2014). Future directions would consider different spatial resolution.

Another type of multi-variate fairness is intersectional fairness where groups are defined by multiple sensitive attributes, e.g., race and gender. Classifiers can be fair when evaluated on independent groups e.g., race and gender independently, but not at their intersections (Buolamwini and Gebru, 2018). The fairness metrics for intersectional fairness are different because the number of groups can be very large. Researchers propose multiple fairness metrics for intersectional fairness that are similar to single group fairness (Kearns et al., 2018; Gopalan et al., 2022; Foulds et al., 2020; Ghosh et al., 2021b). For example, Foulds et al. (2020) propose differential fairness using the idea from differential privacy and p%-rule. It is defined as


$$e^{-\epsilon }\leq \frac{\left(P(\hat{Y}=y|S=s_{i}\right)}{P(\hat{Y}=y|S=s_{j})}\leq e^{-\epsilon }\;\forall s_{i},s_{j}\in S\times S$$

The mitigation algorithms are similar to the single attribute mitigation algorithms described above. However, our approach for assessing them may need to be modified since the number of groups can grow large as the number of intersections grows. Therefore, future work is needed to better understand how to assess bias mitigation methods with larger numbers of groups.

#### 7.2.3 Misclassification distribution on other sensitive attribute inference tasks

In sensitive attribute inference, there are two sources of errors: prediction accuracy and misclassification distribution. This paper mostly focuses on errors from prediction accuracy by studying how sensitive attribute inference models with different levels of accuracy affect the performance of bias mitigation algorithms. We try to understand errors from misclassification distribution by generating three different misclassification errors. However, in real applications, especially with multivariate sensitive attribute, misclassification distributions could be very different. leading to different results. Future work is needed to model the misclassification distribution based on real sensitive attribute inference models in different domains that have less common sensitive attribute distributions.

In general, this work is a first step toward understanding the relationship between accuracy of an inferred sensitive attribute and performance of different bias mitigation approaches. Our results show how uncertainties in the inferred sensitive attribute negatively affect bias mitigation algorithms on various data sets and what types of data bias mitigation algorithms are more sensitive to uncertainties (lower accuracies) in the inferred sensitive attribute. Our results also suggest that using an inferred sensitive attribute is a reasonable approach for reducing the bias of a machine learning algorithm when a ground truth one is not available, opening the door for improving fairness of black box AI systems.

## Data Availability

The original contributions presented in the study are included in the article/Supplementary material, further inquiries can be directed to the corresponding author.

## Footnotes

^1^We acknowledge that there are more than two possible values for gender and race. We note that in the two data sets that use gender as a sensitive attribute (credit card client data and Wikidata), we only have binary information as ground truth data and therefore, can only infer binary sensitive attribute values. In the third data set, where the sensitive attribute is race (COMPAS data), the original fairness study published by the data creators investigated unfair treatment between white and black defendants (Jeff et al., 2016) and major fairness toolkits, such as Fairlearn (Bird et al., 2020) and AIF360 (Bellamy et al., 2019), use binary race (white and non-white) as the sensitive attribute. While not ideal, we believe it is important to first investigate the binary version of our problem. We leave the multi-value version of the problem for future work, discussing it briefly in Section 7.

^2^ https://github.com/GU-DataLab/frontier_sensitive_attribute

^3^In cases where there is not a disadvantaged group, we select the class label that occurs less frequently in the data set to be the minority group.

^4^We acknowledge that there are other distributions that could be tested. We confine this study to three that clearly capture different dynamics for the sensitive attribute groups.

^5^We note that in additional to 0.75 balanced accuracy in inferred sensitive attribute, we conduct these experiments with a balanced accuracy at 0.7 and 0.8. We found that the difference in fairness score is linear to the difference in the balanced accuracy in inferred sensitive attribute.
