# Supplementary material for: Impact on bias mitigation algorithms to variations in inferred sensitive attribute uncertainty
Source: Front Artif Intell. 2025 Mar 6;8:1520330. doi: 10.3389/frai.2025.1520330 (PMC11924408; doi:10.3389/frai.2025.1520330)
Supplement: Supplementary file 1 [file Data_Sheet_1.pdf]

Appendix A. Simulation results on COMPAS and credit card client data

Figure 1, 2, and 3 show the outcome prediction model performance and fairness for the COMPAS data set. Figure 4, 5, and 6 show the outcome prediction model performance and fairness for the credit card data data set.

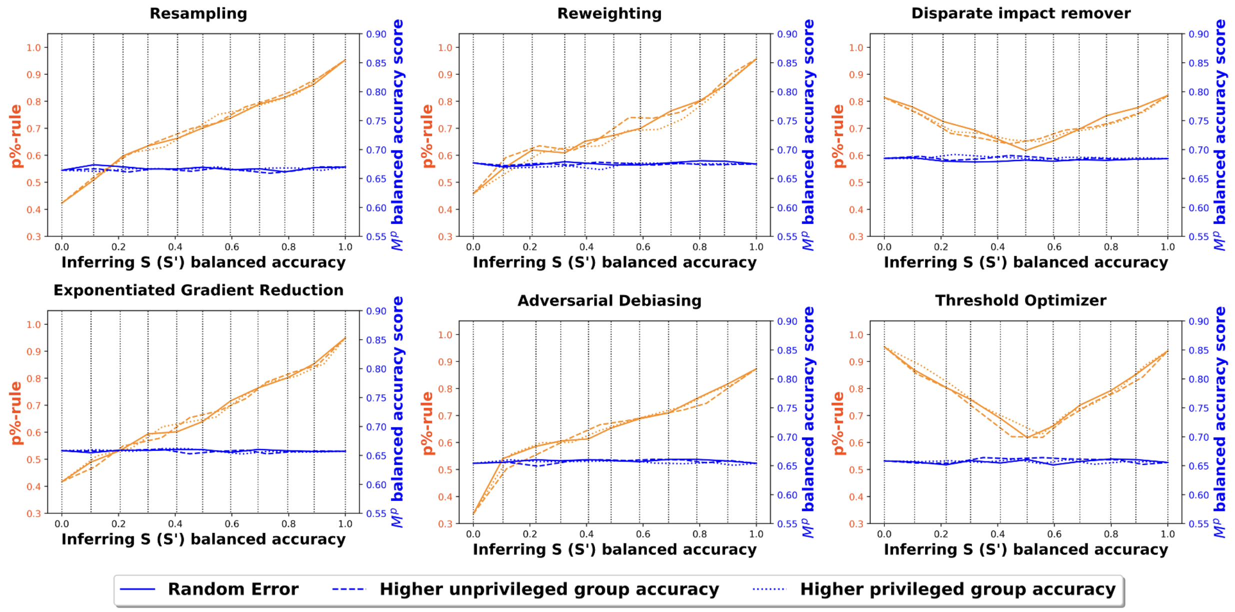

Figure 1: Balanced accuracy and fairness scores of the outcome prediction models using COMPAS data with logistic regression prediction model

Appendix B. Performance and fairness results on Wikidata with logistic regression, SVM and deep learning model with attention mechanism

Table 4, 2 and 3 show the outcome prediction model performance and fairness for the Wikidata set with logistic regression, SVM and deep learning outcome prediction model. For each outcome prediction model, the accuracy and fairness scores are similar across the three gender inference models (M^S). In the deep learning model with DIR mitigation algorithm, the balanced accuracy score is lower than all other mitigation algorithms. One possible reason is that DIR changes feature values to remove the correlation between training features and the sensitive attribute. However, the features here are text embedding vectors from BERT model and changing the embedding vector values could change the semantic representation and hurt the model performance.

Table 1: Balanced accuracy and fairness score of the outcome prediction model using inferred sensitive attribute on Wikidata with logistic regression prediction model

	Balanced accuracy			p%-rule		
	Clip image	BERT emoji	BERT text	Clip image	BERT emoji	BERT text
RS	0.82	0.823	0.825	0.687	0.691	0.697
RW	0.822	0.828	0.827	0.679	0.681	0.674
DIR	0.821	0.822	0.821	0.704	0.701	0.698
AD	0.822	0.821	0.819	0.674	0.679	0.678
EGR	0.811	0.812	0.815	0.734	0.742	0.723
TO	0.821	0.817	0.819	0.702	0.709	0.696

Table 2: Balanced accuracy and fairness score of the outcome prediction model using inferred sensitive attribute on Wikidata with SVM prediction model

	Balanced accuracy			p%-rule		
	Clip image	BERT emoji	BERT text	Clip image	BERT emoji	BERT text
RS	0.772	0.775	0.775	0.668	0.674	0.648
RW	0.771	0.772	0.779	0.652	0.653	0.643
DIR	0.779	0.775	0.776	0.702	0.699	0.67
AD	0.767	0.77	0.768	0.692	0.687	0.671
EGR	0.763	0.765	0.768	0.668	0.663	0.651
TO	0.768	0.771	0.769	0.706	0.687	0.695

Table 3: Balanced accuracy and fairness score of the outcome prediction model using inferred sensitive attribute on Wikidata with deep learning prediction model

	Balanced accuracy			p%-rule		
	Clip image	BERT emoji	BERT text	Clip image	BERT emoji	BERT text
RS	0.829	0.831	0.833	0.662	0.678	0.668
RW	0.825	0.828	0.826	0.663	0.67	0.657
DIR	0.792	0.798	0.796	0.722	0.718	0.708
AD	0.821	0.817	0.811	0.652	0.664	0.648
EGR	0.823	0.818	0.821	0.652	0.66	0.651
TO	0.828	0.813	0.815	0.649	0.663	0.65

Table 4: Standard error on balanced accuracy and fairness score of the outcome prediction model using inferred sensitive attribute on Wikidata with logistic regression prediction model

	Balanced accuracy			p%-rule		
	Clip image	BERT emoji	BERT text	Clip image	BERT emoji	BERT text
RS	0.021	0.018	0.023	0.019	0.016	0.024
RW	0.019	0.017	0.018	0.022	0.016	0.017
DIR	0.026	0.024	0.821	0.028	0.019	0.02
AD	0.026	0.029	0.027	0.019	0.02	0.017
EGR	0.025	0.026	0.024	0.019	0.022	0.018
TO	0.029	0.026	0.025	0.023	0.021	0.023

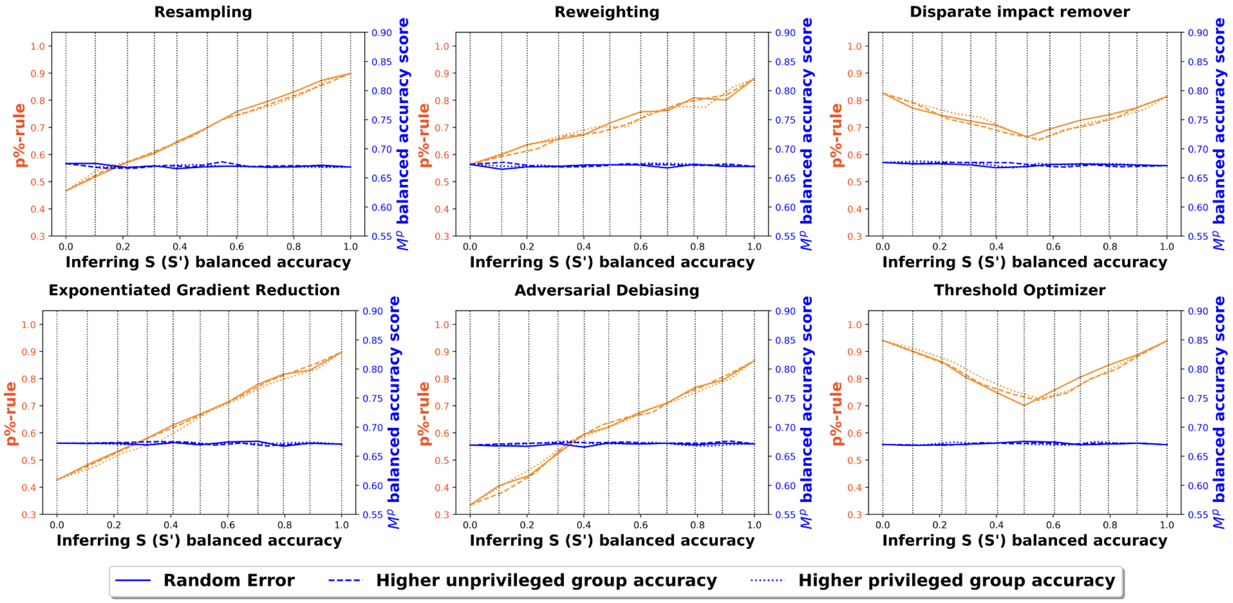

Figure 2: Balanced accuracy and fairness scores of the outcome prediction models using COMPAS data with SVM prediction model

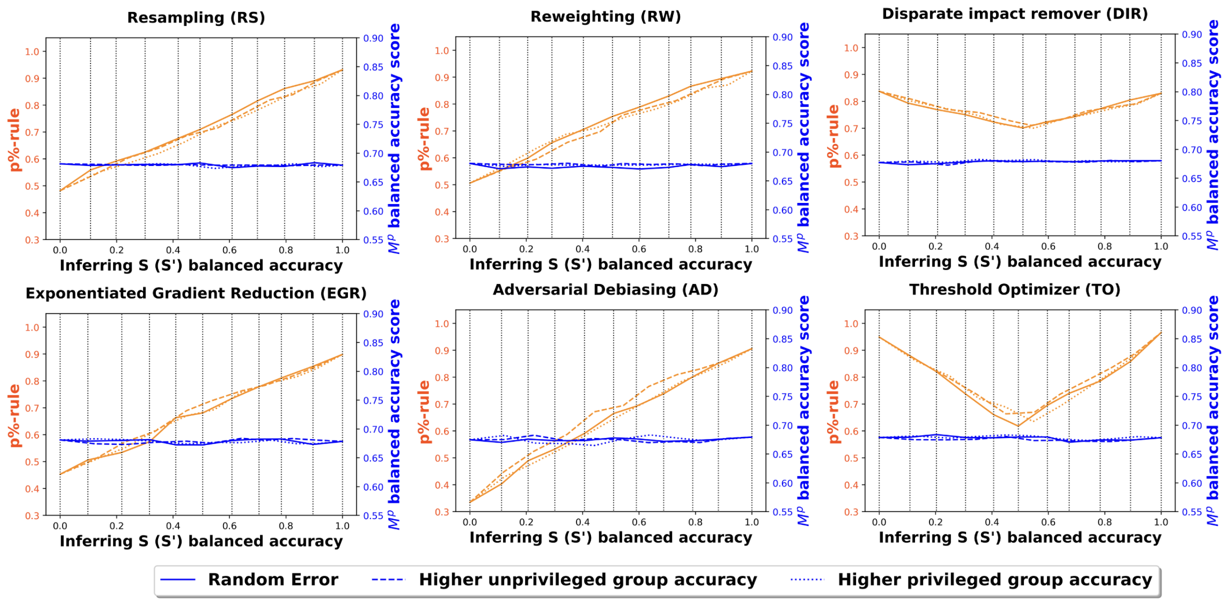

Figure 3: Balanced accuracy and fairness scores of the outcome prediction models using COMPAS data with neural network prediction model

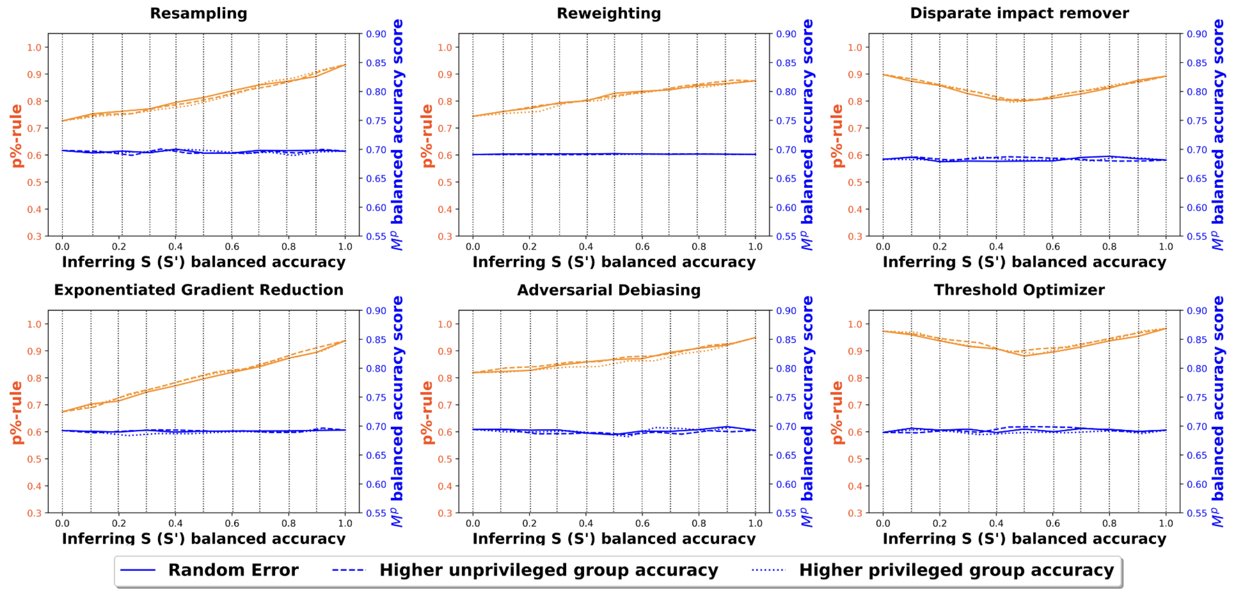

Figure 4: Balanced accuracy and fairness scores of the outcome prediction models using credit card client data with logistic regression prediction model

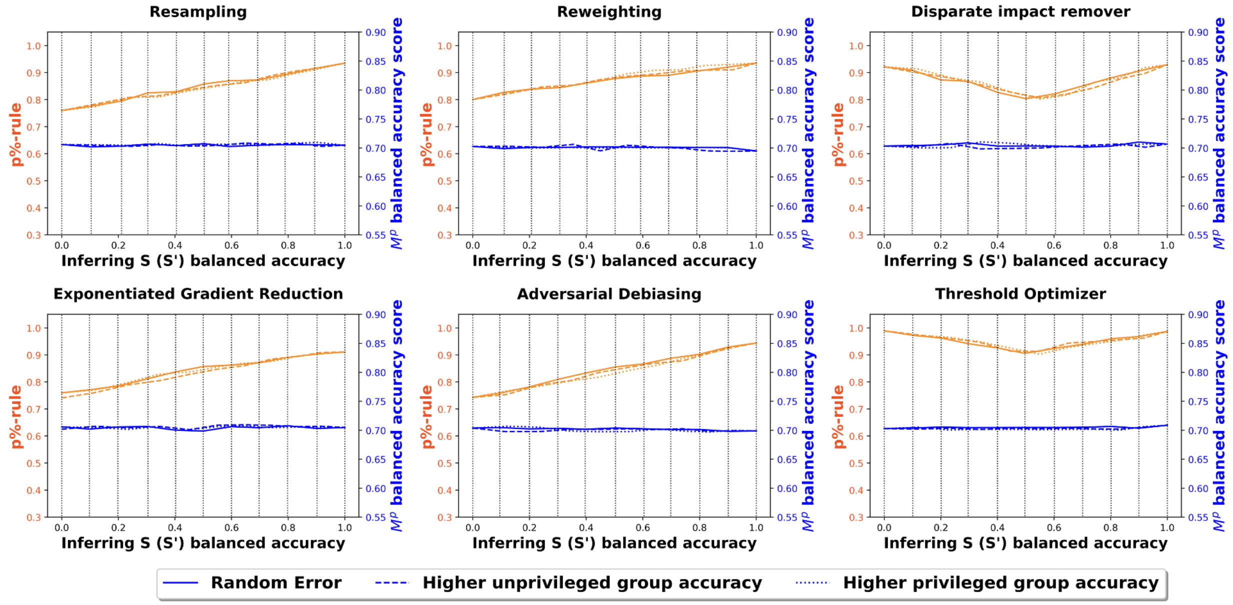

Figure 5: Balanced accuracy and fairness scores of the outcome prediction models using credit card client data with SVM prediction model

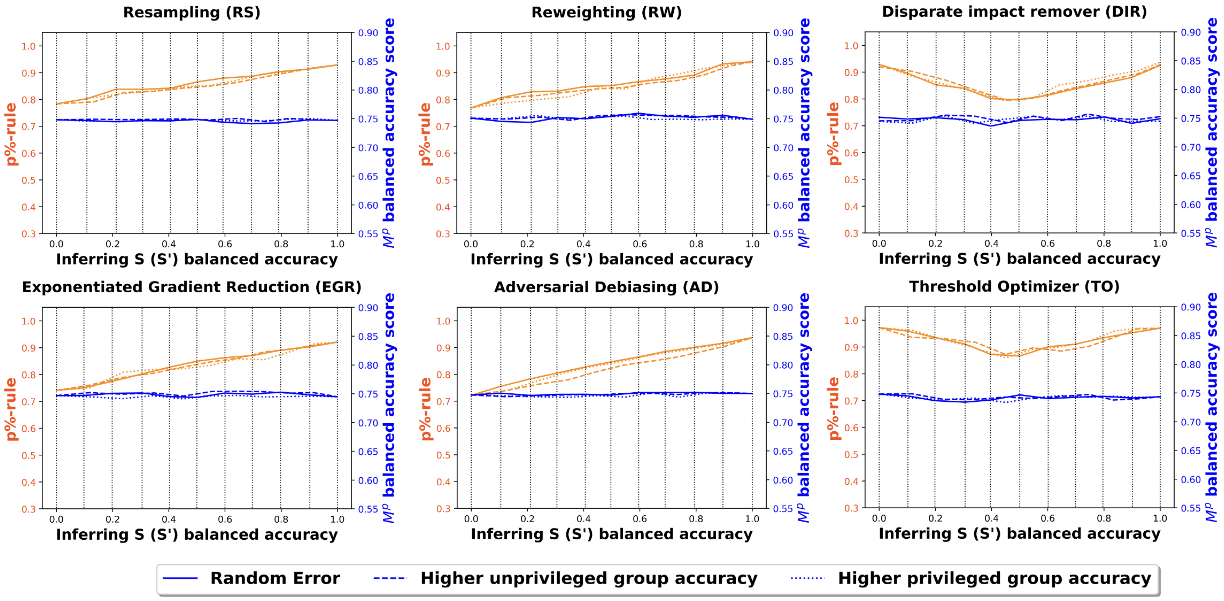

Figure 6: Balanced accuracy and fairness scores of the outcome prediction models using credit card client data with neural network prediction model
